# Hypnic Headache Responds to Topiramate: A Case Report and a Review of Mechanisms of Action of Therapeutic Agents

**DOI:** 10.7759/cureus.13790

**Published:** 2021-03-09

**Authors:** Hassan Kesserwani

**Affiliations:** 1 Neurology, Flowers Medical Group, Dothan, USA

**Keywords:** headache disorders, medication therapy management

## Abstract

Hypnic headaches are unique as they are exclusively nocturnal, occurring in rapid-eye movement (REM) and non-REM sleep. Their nocturnal nature suggests a role for cyclic mechanisms involving the hypothalamus despite conflicting imaging results for the role of the posterior hypothalamus. Nevertheless, pharmacological therapeutics acting as highly effective agents, such as caffeine and melatonin, can modulate the sleep-wake cycle. In addition, indole agents such as indomethacin that are anti-nociceptive and affect cerebral blood flow also prove to be efficacious. Gabanoids and topiramate also have reported efficacy. We report the case of a topiramate-responsive hypnic headache patient and outline in detail the potential mechanisms of topiramate and the other therapeutic agents and adumbrate on the neuronal networks of migraine, the trigeminal autonomic cephalgias and suggest a potential neural circuit for hypnic headaches.

## Introduction

Hypnic headaches are sleep-induced nocturnal headaches that are usually holocranial but can be bifronto-temporal and rarely bioccipital, dull and moderate-to-severe in intensity. Migrainous symptoms such as nausea, photophobia, and phonophobia may occur in 30% of patients. Some hypnic headaches can be associated with mild autonomic symptoms such as rhinorrhea or lacrimation. The average duration of the headache is 90 minutes with a mean frequency of 21 days per month. There is sexual dimorphism; approximately two-thirds of patients are women and one-third are men. Three-quarters of patients go into remission with caffeine, lithium, melatonin, or indomethacin [1]. There have also been isolated case reports of therapeutic responses to acetazolamide, topiramate, and the gabanoids (gabapentin and pregabalin) [2-4].

The International Headache Society (IHS) defines hypnic headaches as: 1) recurring headache attacks; 2) occurring exclusively during sleep and resulting in awakening; 3) lasting at least three months with a frequency of more than 10 headache days per month; 4) each attack lasts from 15 minutes to 4 hours; 5) no autonomic symptoms or restlessness [5]. However, criterion (5) may be seen in some patients.

The hypnic headaches are a diagnosis of exclusion as rare cases of cerebellar hemangioblastomas, pituitarymacro adenomas, and brainstem lesions present with similar symptomatology [6]. 

A vortex-based morphology study in 14 hypnic headache patients revealed reduced posterior hypothalamus volume. This is concordant with the nocturnal nature of hypnic headaches, and their response to agents that are effective for cyclic disorders such as lithium (bipolar disease) and melatonin (sleep-wake cycle) [7]. These findings concur with the idea that the hypothalamus is part of the central autonomic network, involved in the control of homeostasis and pain. It is connected with the peri-aqueductal gray matter (pain) and the locus coeruleus and median raphe nuclei, both are which are involved in the sleep-wake cycle. 

Hypnic headaches were thought to be a disorder of rapid-eye-movement (REM) sleep, as they usually occur between three-to-four o'clock in the morning and when patients awaken from REM sleep [8-10]. REM sleep is also associated with sympathetic discharge which is also associated with the posterior hypothalamus [11]. However recent studies have shown that hypnic headaches can also occur during non-REM sleep [12].

## Case presentation

We present the case of a 48-year-old woman with a four-month history of exclusive nocturnal bilateral occipital pain that awakens her at 11 pm after falling asleep at 8 pm. The pain occurs every night and is described as a pressing intense pain that can last up to three hours. It is associated with nausea but no photophobia, phonophobia, or autonomic symptoms. Treatment with meloxicam 7.5 milligrams (mg) and diclofenac 75 mg nightly did not help. There is no history of sleep apnea. Past medical history is significant for hypertension treated with lisinopril 10 mg daily. 

On examination, blood pressure (BP) is 113/75 mmHg with a pulse of 83 beats/minute. Height is 5 foot and 4 inches with a weight of 123 pounds and a body-mass-index (BMI) of 21.1. Pertinent neurological examination reveals full ocular motion with symmetric-reactive pupils and normal accommodation. An ophthalmological examination revealed no papilledema. Ptosis is absent. The rest of the cranial nerve examination and neurological examination is normal. Noteworthy is the absence of occipital tenderness over the occipital groove, which may be a sign of occipital neuralgia. Furthermore, neck extension or rotation does not elicit occipital pain over the course of the occipital nerve.

**Figure 1:**
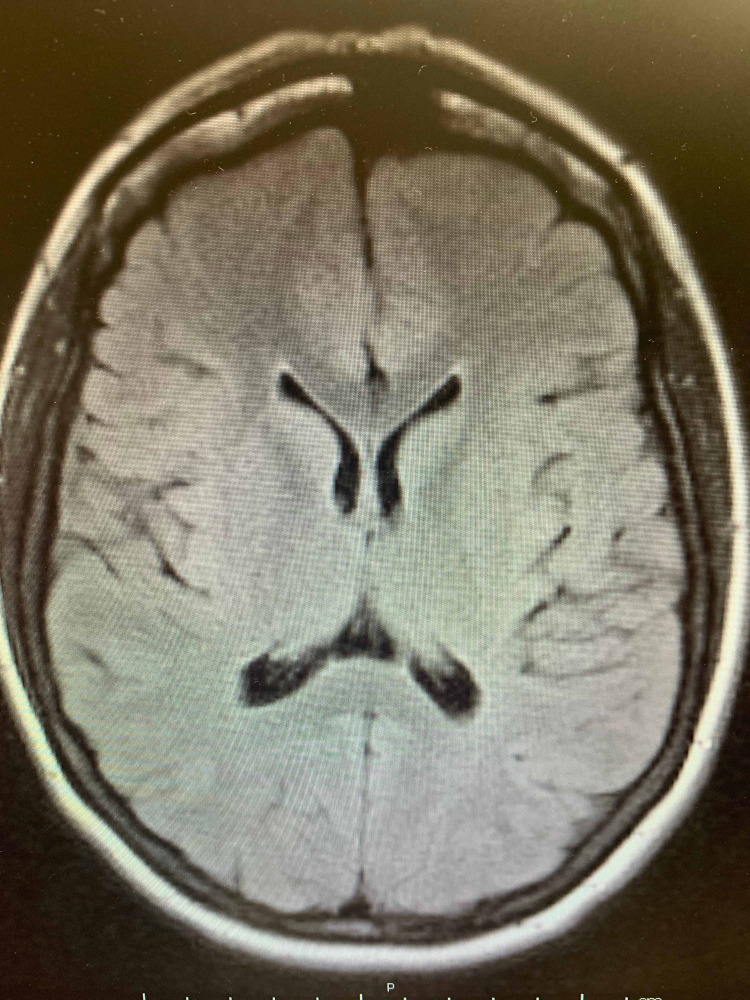
FLAIR MRI: normal brain. FLAIR-MRI brain: fluid-attenuated inversion recovery magnetic resonance imaging of the brain

An MRI examination of the brain was normal, ruling out a structural lesion of the brain (Figure 1).

Hypnic headaches were diagnosed, noting that less than 10% of patients present with occipital headaches [1]. Occipital neuralgia was excluded by the lack of sharp occipital pains, lack of tenderness over the occipital groove, and the absence of occipital pain with neck extension or rotation. The lack of exacerbation of the headache with the Valsalva maneuver, as in coughing, rules out a primary cough headache syndrome. The normal MRI brain rules out cerebellar ectopia. None of the IHS criteria for migraine are met.

The patient was treated with a weekly escalating dose of topiramate 25 mg nightly with a target dose of 100 mg nightly and there was near-complete resolution of headaches by eight weeks, with rare mild breakthrough occipital headaches and without any side-effects. The patient opted out of a trial of caffeine due to the risk of insomnia and indomethacin was not favored by the patient due to risk of peptic ulceration, hypertension, and the possibility of renal dysfunction. 

## Discussion

Hypnic headaches are usually amenable to therapy with a wide range of agents. The agents used generally affect sleep and the circadian rhythm (caffeine, melatonin, and lithium), affect vasodilatation (indomethacin) or alter neuronal excitability and affect sleep (gabanoids, topiramate). Interestingly both melatonin and indomethacin harbor an indole backbone, an aromatic heterocyclic ring which may explain their similar action in pain modulation. The postulated mechanisms of action of the various agents and some practical tips are listed below (Table 1) [6, 13]. 

**Table 1:** Therapeutic agents with hypothetical mechanisms of action. GABA, gamma-amino butyric acid

Agent	Proposed mechanism of action	Noteworthy facts
Caffeine	Anti-nociceptive: adenosine 2-A, 2-B antagonist, peripheral action; influences sleep	First line agent for hypnic headaches; dose not standardized. Cup of coffee (200 mg caffeine), cup of brewed tea (70 mg caffeine). Caffeine tablets are 100, 200 mg.
Indomethacin	Crosses blood brain barrier; inhibits nitric oxide induced dural vasodilation	Dosage 25-150 mg nightly; 70% response rate
Lithium	Inhibits circadian clock	Withdrawal after chronic lithium treatment may lead to hypnic headaches. Start at 150 mg nightly; may titrate up to 600 mg
Melatonin	Melatonin receptors in the suprachiasmatic nucleus of the hypothalamus; may enhance GABA signaling and inhibit nitric oxide	3-12 mg nightly
Gabapentinoids (gabapentin, pregabalin)	Alfa 2-delta calcium channel modulators; may act on pain pathways and sedating effect	Gabapentin 300 mg qhs, pregabalin up to 75 mg qhs
Topiramate	Sodium channel blocker, GABA potentiation, glutamate antagonism; may reduce network excitation	Usually low doses, up to 100 mg

In addition to sodium channel blockade, gamma-amino butyric acid (GABA) potentiation, and glutamate antagonism, topiramate also has carbonic anhydrase inhibitor activity [14]. It is also interesting that topiramate may also reduce neuropeptide-Y (NPY) and the fat/mass-obesity associated protein in the ventro-medial nucleus of the hypothalamus, which is part of the posterior hypothalamus [15]. 

In inflammatory animal models, topiramate has been shown to reduce hyperalgesia through central GABA-A and opioid agonism and by peripheral alfa-2 adrenergic agonism [16]. 

There is also functional magnetic resonance imaging (fMRI) evidence that a single dose of 100 mg of topiramate can reduce activity of thalamacortical pain pathways by decoupling the thalamus from the somatosensory cortex, posterior cingulate cortex, and precuneus [17]. 

The central nervous system (CNS) harbors at least nine different isoforms of carbonic anhydrase, where it is ubiquitous. It is one of the most basic enzymes in nature, catalyzing the reversible reaction between water and carbon dioxide, producing bicarbonate and proton ions, and control of acid-base balance. Besides its anti-convulsant properties, carbonic anhydrase inhibitors including topiramate reduces intracranial pressure, acts as a vasodilator as well as reducing neuropathic pain and cerebral ischemia. The pervasive and protean actions of the carbonic anhydrase family has found use in the treatment of a wide range of neurological diseases such as pseudotumor cerebri (idiopathic intracranial hypertension), idiopathic cough headaches, acute mountain sickness, menstrual migraine, catamenial epilepsy, periodic paralysis, and episodic ataxias [18]. Topiramate's use and effectiveness in hypnic headaches makes intuitive sense, but we do have simpler options like caffeine (a cup of coffee before bedtime) and melatonin.

On a final note, it is now well established that the neuronal network for migraine pathogenesis is the trigeminal system (trigeminal nucleus caudalis and trigemino-thalamo-cortical pathways) which correlates beautifully with the trigemino-vascular hypothesis. Similarly, the network for the trigeminal autonomic cephalgias, hemicrania continua, and cluster headaches, is the pathway from the trigeminal nucleus to the sphenopalatine ganglion and greater superficial petrosal nerve via a relay at the superior salivatory nucleus [19]. 

**Figure 2:**
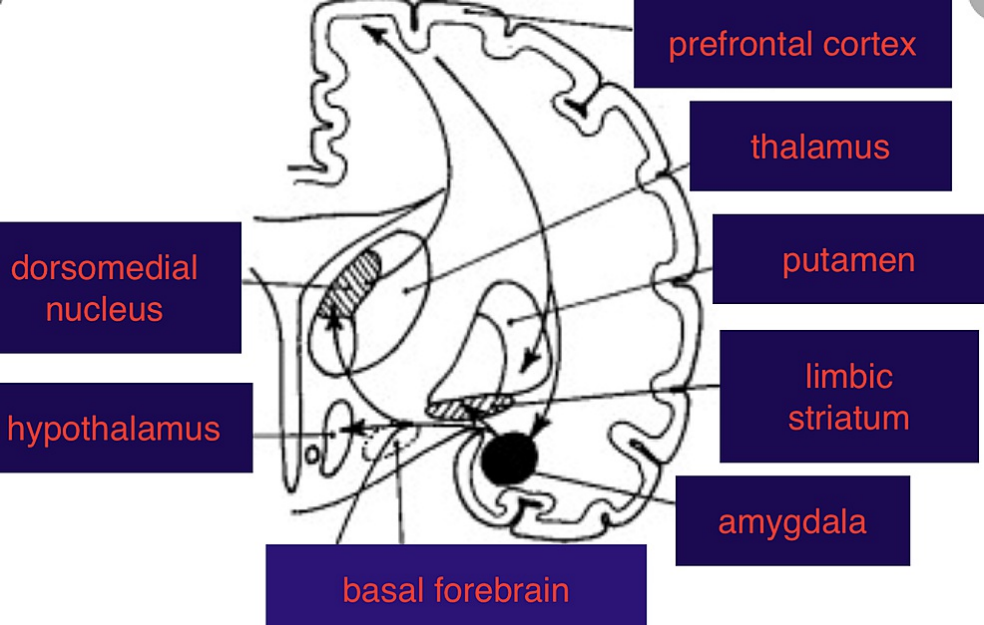
Proposed neural circuitry for hypnic headaches: prefrontal cortex, thalamus, hypothalamus, and basal forebrain. This circuit is involved in pain and sleep-wake cycle regulation.

We propose that the hypnic headache pathway involves the wake-promoting sleep network which includes the thalamus, hypothalamus, and basal forebrain [20]. This explains the nocturnal nature of the headaches, activation of the thalamus (pain and proximity to hypothalamus), and efficacy of sedating and circadian rhythm-modulatory agents. The basal forebrain is rich in acetylcholine and there are anecdotal reports of the benefits of anti-cholinergics, such as amitryptyline [6]. Topiramate, known to have an anti-convulsant effect, may act on the amygdala, melatonin at the hypothalamus, pregabalin and gabapentin also have anti-convulsant activity and pain modulatory effects and may act at the amygdala and thalamus respectively. This is speculative and the neuronal circuit is predicted to involve the prefrontal cortex, thalamus, hypothalamus, amygdala, and basal forebrain (Figure 2).

Further functional MRI studies capturing a headache-in-evolution will clarify the neuronal circuitry. It is important to note that this circuit is distinct from the trigemini-vascular network and the network for trigemini-autonomic cephalgia. 

## Conclusions

Hypnic headaches are distinct from migraine and the trigeminal autonomic cephalgias. Their exclusive nocturnal occurrence is a useful diagnostic clue and their response to caffeine and melatonin is noteworthy. Thinking of the various primary headache syndromes as network dysfunctions helps us understand their semiology and pharmacological treatment options. In this article we collate and summarize the latest pertinent clinical and pathophysiological finding of this unique primary headache disorder and emphasize the role of topiramate in its therapeutics. 
